# New Method for Reduced-Number IMU Estimation in Observing Human Joint Motion

**DOI:** 10.3390/s23125712

**Published:** 2023-06-19

**Authors:** Thang Hoang, Yaojung Shiao

**Affiliations:** 1 Faculty of Transportation Mechanical Engineering, The University of Danang-University of Science and Technology, Danang 550000, Vietnam; hthang@dut.udn.vn; 2 Department of Vehicle Engineering, National Taipei University of Technology, Taipei 106344, Taiwan; 3 Railway Vehicle Research Center, National Taipei University of Technology, Taipei 106344, Taiwan

**Keywords:** joint motion, motion recognition, Inertial Measurement Unit (IMU)

## Abstract

Observation of human joint motion plays an important role in many fields. The results of the human links can provide information about musculoskeletal parameters. Some devices can track real-time joint movement in the human body during essential daily activities, sports, and rehabilitation with memory for storing the information concerning the body. Based on the algorithm for signal features, the collected data can reveal the conditions of multiple physical and mental health issues. This study proposes a novel method for monitoring human joint motion at a low cost. We propose a mathematical model to analyze and simulate the joint motion of a human body. The model can be applied to an Inertial Measurement Unit (IMU) device for tracking dynamic joint motion of a human. Finally, the combination of image-processing technology was used to verify the results of model estimation. Moreover, the verification showed that the proposed method can estimate joint motions properly with reduced-number IMUs.

## 1. Introduction

**Figure 1:**
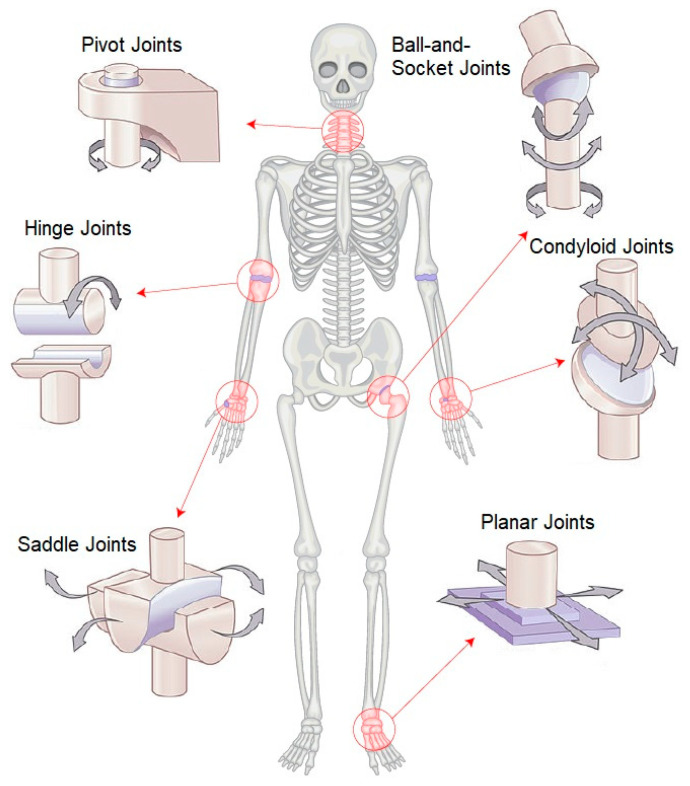
Synovial Joint Classifications: Types and Examples.

Monitoring the motion of the skeleton is important for predicting the risk of diseases in humans and is beneficial for therapists and physicians. The several types of inflammatory diseases [1] affect nearly all joints [2], and people of all ages can suffer from arthritis-related issues. The human framework, a good mechanical structure, consists of different bones, and joints link bones and provide stability and mobility to the skeleton. A human body contains three types of joints: fibrous (immovable), cartilaginous (semi-movable), and synovial (freely movable) [3]. Synovial joints are important joints of the body because they provide mobility by allowing load-bearing, low-friction, and wear-resistant smooth movement between articulating bone surfaces [4]. A total of six groups of synovial joints exist in the body. The six groups of synovial joints in the human body are pivot, hinge, condyloid, saddle, ball-and-socket, and plane joints, as depicted in Figure 1. Each synovial joint has a distinct structure, purpose, and extent of mobility. The hinge joint, for example, permits movement in only one plane, whereas the ball-and-socket joint allows movement in numerous planes. Monitoring synovial joint mobility is critical for identifying and controlling joint-related issues such as osteoarthritis, rheumatoid arthritis, and other inflammatory joint illnesses [5,6]. Understanding the various kinds of synovial joints and their roles allows clinicians and doctors to create successful therapy strategies for patients with joint-related illnesses [7].

**Figure 2:**
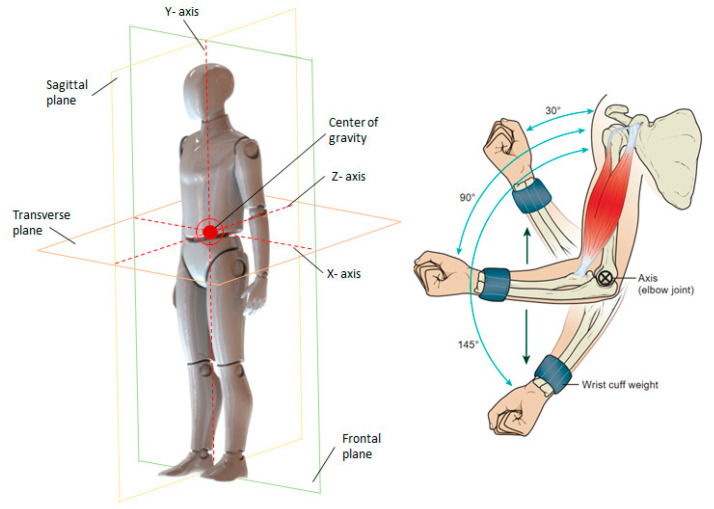
Cardinal planes of motion and plane of human movement.

The study of human motion is called kinesiology, and combines data with modern technology to create a highly sophisticated means for analyzing human movement. The body and its segments move in planes of motion, called the cardinal planes of motion, around respective axes (Figure 2). These planes rotate around x, y, and z axes. As depicted in Figure 2, the x or medial–lateral axis runs side to side and is located in the frontal plane; the y or vertical axis runs up and down or superior–inferior and is in a transverse plane; and the z or anterior–posterior axis runs from front to back and is in the sagittal plane 2. All movements occur along a plane of motion and around the axis of that motion.

**Figure 3:**
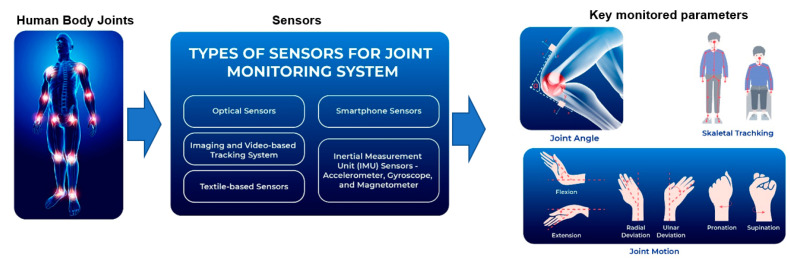
Joint monitoring sensor technologies and monitored parameters.

By utilizing tracking devices, we can collect data on the dynamic joint motion of humans, enabling the development of various applications such as fall detection systems, elderly monitoring, gait pattern and posture analysis, and pedestrian navigation. These applications play a crucial role in recognizing human activities and have implications for enhancing safety and well-being. The human activity-recognition algorithm is similar to an all-purpose pattern-recognition system and corresponds to a collection of steps from data collection to activity classification [8]. It is often divided into two approaches supported by machine learning techniques: shallow algorithms (e.g., Support Vector Machine, K-Nearest Neighbors, and call tree) and deep algorithms (e.g., Convolutional Neural Network, Recurrent Neural Network, Restricted Boltzmann Machine, Stacked Autoencoder, and Deep Feedforward Network) [9,10]. These approaches can be distinguished based on the method used for data extraction, whether manually or mechanically. This process involves transformation of the information extracted from the sensors for developing economic classification models for human activities. Therefore, recently, the development of methods for tracking motion in humans has been extensively researched [11,12,13]. Many techniques have been proposed for joint monitoring. Figure 3 shows the methods used for monitoring a human joint.

The data fusion technique is used for observing objects that can enable feature extraction. The combination of an accelerometer, gyroscope, and magnetometer is called an Inertial Measurement Unit (IMU) and is used for measuring the angular velocity and position of the object in the Cartesian coordinate system. In addition, some commercially available IMU sensors have functions that can be demonstrated for the rotation matrix, quaternion, or Euler angles. In many studies, more than two IMUs have been used for monitoring human joints [13,14,15,16,17]. Based on the fusion data obtained from an IMU, an algorithm was used to present the dynamics of the joint angle. In 2008, Favre and Jolles used two IMUs mounted on legs to analyze knee angle [16]. In 2011, Saba Bakhshi developed a tracking joint for two legs with four IMUs [15]. An algorithm was developed for an IMU for estimating the position and accuracy of the sensor. The attitude and heading reference systems (AHRSs) are algorithms used for solving the problem of orientation measurement relative to the direction of gravity and the Earth’s magnetic field on the IMU. The AHRS applies a Kalman filter to provide an optimal least mean variance estimation [18]. This results in high accuracy for each calculated state because of the combined measurements from different sources. However, the AHSR method is costly, and equal efficiency is desired. A complementary filter was developed to address this problem. The gyroscope and accelerometer data are a feasible dynamic attribute and good static feature for high- and low-frequency attitude estimations, respectively. The gradient descent-based complementary (GDC) and explicit complementary filter (ECF) algorithms are the latest advancements in complementary filters [19,20]. Both techniques use quaternions to represent rotation. Although GDCA and ECF have a highly effective and novel approach in terms of low-cost attitude estimation, ECF is slightly more accurate than GDCA [21]. In addition, ECF is applied to a system with limited resources, and a framework is designed to integrate multisensory data in the context of navigation in Kalman filtering variants, among which the extended Kalman filter (EKF) performs the best for non-linear plant models, as usually encountered in navigation [22]. In our study, we developed a new method for reducing the number of IMU matches on the body to track human joints and used the EKF for estimating the accuracy of the sensor in determining the position.

## 2. The Applications of IMU Sensor in Observing Motion

### 2.1. Estimation of Sensor Fusion

**Figure 4:**
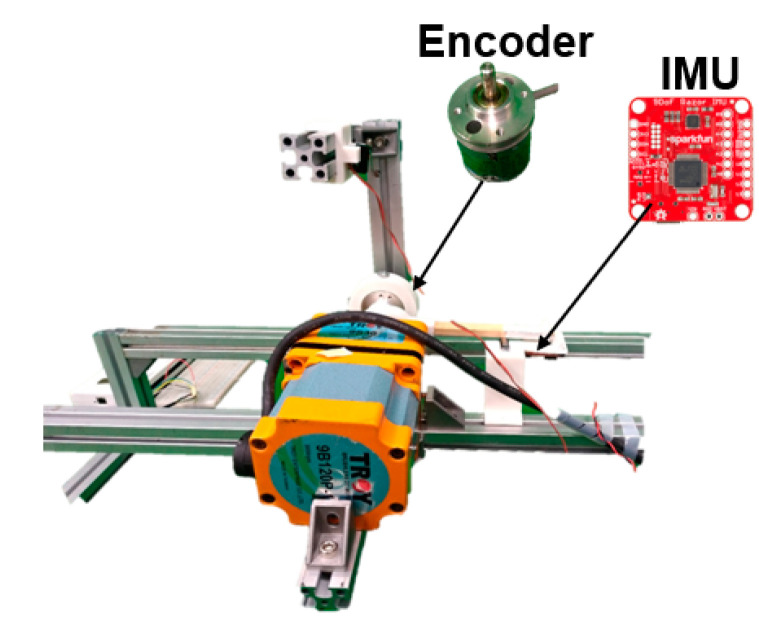
The model tests the estimation algorithms on the IMU sensors.

In order to address the inherent errors present in the raw IMU data [23], we employed estimation algorithms to mitigate the noise. To obtain more accurate angles, a specialized apparatus consisting of a rotary encoder and a motor (depicted in Figure 4) was utilized in this research to quantify angle errors. The encoder data served as a benchmark reference for calculating the root mean square error. By securely attaching the Inertial Measurement Unit (IMU) to the motor’s rotational axis and strategically placing switches at both the vertical and horizontal axes of rotation, the IMU would automatically reverse its direction upon contact with the upper switch and promptly come to a stop upon interacting with the lower switch. This ensured a consistent rotation speed during data collection, enhancing the reliability of the measurements.

**Figure 5:**
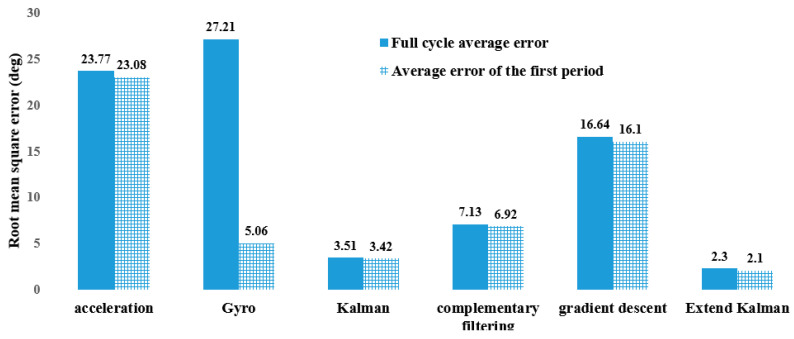
Comparison of root mean square errors among estimation algorithms.

We applied various algorithms to estimate the accuracy of the angle, including acceleration estimation, gyro estimation, Kalman filter, Complementary filter, gradient descent, and Extended Kalman filter. Subsequently, we found that the Extended Kalman filter yielded the best results, as depicted in Figure 5.

### 2.2. Zero-Velocity Detector

An IMU sensor is known to contain considerable noise [24]. From the mechanical phenomenon detector data of gyroscopes and accelerometers, a slight offset can be observed within the average signal output, even in the absence of movement. This can be called the sensor bias. The physical properties of these sensing elements change over time, resulting in many characteristics over time [25]. Internal sensor biases can increase depending on sensor usage and time. In the absence of bias correction and use of gyroscopes solely for orientation estimate, the orientation estimate would “drift” owing to the sensor bias. Moreover, the limitation of standard zero-velocity detectors is their threshold-based activation, given a fixed threshold, and the detectors fail to perform reliably across a variety of gait motions. There are five detectors: stance hypothesis optimal estimation, angular rate energy detector, acceleration-moving variance detector, memory-based graph theoretic detector, and VICON stance detection [26]. We used the stance hypothesis best estimation (SHOE) detector, which is predicated on a generalized chance quantitative relation check (GLRT) that indicates moving IMU. Table 1 provides an explanation of the parameters used in formula (1). If the likelihood falls below a threshold, γ, the hypothesis of the IMU being stationary is accepted (meaning that the particular force measured is strictly due to gravity, which has an angular rotation rate of zero) [27].

(1) $${iszv}_{k}=T_{k}\left(a,\omega \right)=\frac{1}{W}\sum_{n=k}^{k+W-1}(\frac{1}{\sigma_{a}^{2}}{\left\|a_{n}\right.}^{}-g\frac{\bar{a}}{\left\|\right.\bar{a}\left.\right\|}{\left.\right\|}^{2}+\frac{1}{\sigma_{\omega }^{2}}\left\|\right.\omega_{n}{\left.\right\|}^{2})\leq \gamma$$

### 2.3. Extended Kalman Filter

We used an Extended Kalman Filter (EKF) for motion estimation, which separates the estimated state into non-linear states. Our state consisted of the IMU’s position (*p_k_*), velocity (*v_k_*), and orientation in the quaternion form (*q_k_*) [28,29,30].

$$p_{k}^{T}=\left[p_{x}p_{y}p_{z}\right];$$

$$v_{k}^{T}=\left[v_{x}v_{y}v_{z}\right]$$

$$q_{k}^{T}=\left[q_{0}q_{1}q_{2}q_{3}\right]$$

**Figure 6:**
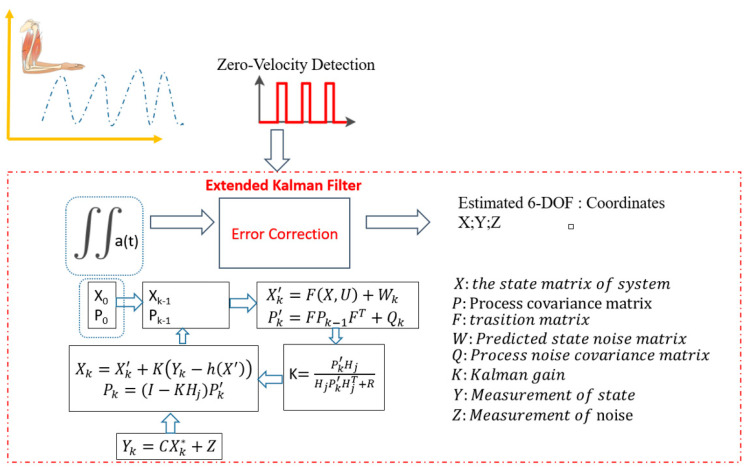
The diagram algorithm of EKF with zero-velocity detection.

The nonlinear state equation propagates the nominal state over time by integrating the IMU outputs:

$$x_{k}=\left[p_{k}^{T}v_{k}^{T}q_{k}^{T}\right]$$

(2) $$x_{k}=\left[\begin{array}{c} p_{k}\\ v_{k}\\ q_{k} \end{array}\right]=\left[\begin{array}{c} p_{k-1}+v_{k}∆t+0.5a_{k}{∆t}^{2}\\ v_{k-1}+a_{k}∆t\\ 0.5\Omega (\omega_{k}∆t)q_{k-1} \end{array}\right]$$

$$a_{k}=R_{DMC}*a-[0;0;g]$$

where Δ*t* is the sampling period; *a_k_* is converted from a quaternion representation into a rotation matrix. This means that the local gravity vector is a linear update of the quaternion orientation by an integrated angular rate called Rate Derivative Measurement Correction (RDMC). Figure 6 shows the relationship between zero-velocity detection and EKF. Below the detection of a zero-velocity event, the velocity is (approximately) zero of the observable system. This linear measurement was fused with the error state, updated using the standard EKF model. After adjusting the nominal state based on the error, the error state was reset to zero.

## 3. Novel Model Tracking Joints

### 3.1. Developing a Mathematical Model

**Figure 7:**
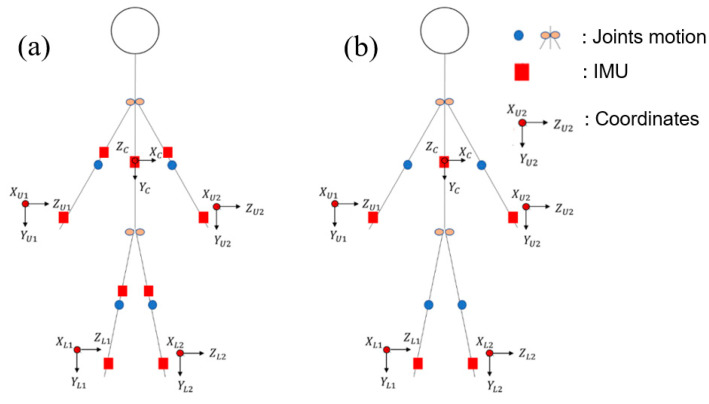
The placement of IMU sensors on the body for kinematic observation. (**a**) Traditional observation; (**b**) The observation model proposed by us.

To accurately capture the movement posture of a human body, it is necessary to consider the displacement that occurs during movement. Traditional motion-capture methods involve the use of cameras and markers placed on the moving body. However, this method is limited to indoor environments and may be affected by occlusion, lighting, and other factors. To overcome these limitations, Inertial Measurement Units (IMUs) have emerged as a promising alternative. These sensors can be attached to various parts of the body and can accurately capture movement in various environments, including outdoor settings. Additionally, they provide real-time data that can be processed quickly and efficiently. Our study focuses on utilizing spatial geometry to calculate angles and displacements. This approach requires the use of an inertial sensor located on the chest, as depicted in Figure 7. The sensor records data such as acceleration and angular velocity, which are then used to calculate the body’s orientation and displacement. One challenge with using IMUs is that they can be costly and may require a large number of sensors to capture all the necessary data. To address this issue, our approach involves optimizing the use of IMUs by minimizing their number. This not only enhances efficiency but also reduces costs while ensuring accuracy. Our method also involves selecting a specific case to study and solve the problem. By doing so, we can gain a more comprehensive understanding of the problem at hand and develop a targeted solution that can be applied to similar cases. To implement our approach, we conducted experiments involving human subjects performing various movements, such as walking and running. The data collected from the IMUs were used to calculate the angles and displacement of the body during these movements. The results showed that our method provided accurate and reliable measurements of body movement.

**Figure 8:**
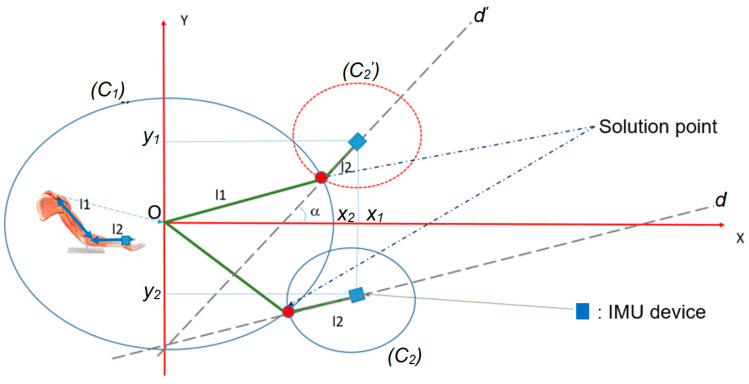
The description of the novel algorithm is based on the geometry in plane coordinates (OXY).

In our research, we aimed to investigate the relationship between muscle contraction and human body length. To achieve this, we proposed several initial hypotheses, with the first being that muscle contraction does not significantly affect the overall length of the human body. Our second hypothesis was that the connection between the arm and body is rotational, rather than translational. To test these hypotheses, we utilized an IMU sensor to determine the position of the center point in the wrist. This allowed us to accurately measure the arm’s motion and gain a better understanding of the rotational relationship between the arm and body. Additionally, we visualized the 3D movement of an object by focusing on each plane separately. In particular, we used the X-Y plane to construct a mathematical model for tracking the arm’s motion. The parameters of geometry in Figure 8 are described in Table 2.

We assumed that the arm was projected onto the X-Y plane, with the center of coordination located at the center point of the shoulder, as illustrated in Figure 6. Using the IMU, we determined the X and Y locations in this plane using the initial position configuration. The trajectory of the arm can be observed when it moves projected onto the X-Y plane. By solving the equation created by the two circles and a line, the location of the elbow can be determined, as shown in Figure 8.

Mathematical identities and notations were used to construct the model, which was then fused with the error state and updated using the EKF standard. Through mathematical modeling, we obtained the system of Equation (3), which is the equation we built up from Figure 8. The elbow location is a solution to this system of equations.

(3) $$\left\{\begin{array}{c} x^{2}+y^{2}=l_{1}^{2}(C_{1})\\ {\left(x-a\right)}^{2}+{\left(y-b\right)}^{2}=l_{2}^{2}(C_{2})\\ y=ax+b(d) \end{array}\right.$$

### 3.2. Simulation of Position of Joints

In this study, LabVIEW was used for simulation. The location of a marked point on the wrist was input. The proposed algorithm was used for determining the location of the elbow. The dynamics of the arm can be simulated based on two different locations on the hinge and saddle joints. We assumed that the location of a point on the saddle joint can be determined from the pixels obtained through image processing using a camera.

**Figure 9:**
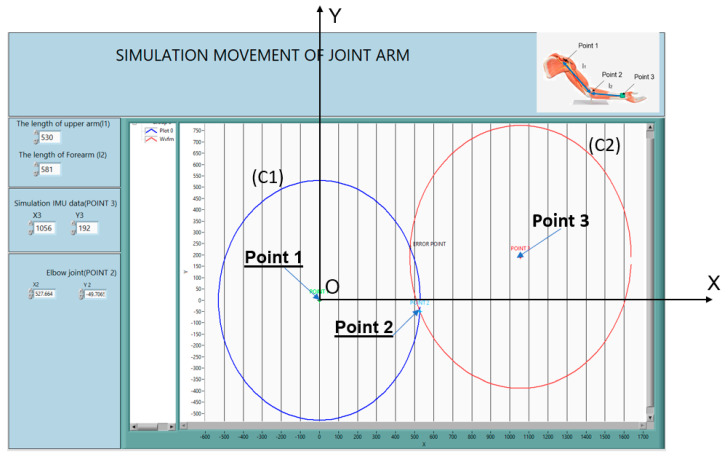
The interface simulation tracking of human joints.

In Figure 9, the intersection of the two circles indicates that solution point 2 is the location of the elbow. Point 1 was assumed to be non-moving. From the third point, we could track the orbit of the hand. In this application, a unit was used as a pixel. One pixel corresponded to 0.042 cm.

### 3.3. Experiment for Verification via Camera

In our research, image processing of the tracking object implemented two pattern-matching methods: pyramidal matching and image understanding (low-discrepancy sampling). Each method used normalized cross-correlation as the core technique. The pattern-matching method consisted of two stages: learning and matching [31,32,33]. First, the formula extracted gray worth and/or edge gradient information from the model image throughout the educational stage. The algorithm then organized and stored the information for facilitating browsing through the examination image. In metal vision, the information learned during this stage was retained as a part of the template image. The pyramid match approximation divided the feature area into more relevant regions using a multidimensional, multiresolution bar graph pyramid. At the highest resolution level of the pyramid, the partitions (bins) are small; at subsequent levels, their sizes increase until one segment covers the entire feature. Two points from any two-point set can begin to share a bin at some point along this gradation in bin size, and once they do, they are considered matching. When points in a bin are regarded as matched, the scale of that bin displays the largest distance between any two issues, allowing us to extract an identical score without having to compute the distances between each of the points in the input sets. For an input set *X*, the feature extraction function *Ψ* is defined as Equation (4):

(4) $$\psi \left(X\right)=\left[H_{0}\left(X\right),\ldots ,H_{L-1}\left(X\right)\right]$$

where $X\in S,L={log}_{2}D+1,H_{i}\left(X\right)$ is a histogram vector formed over points in *X* using dimensional bins of length 2i, $\psi \left(X\right)$ is a histogram pyramid, and $H_{i}\left(X\right)$ has dimension $r_{i}={\left(\frac{D}{2^{i}}\right)}^{d}$.

**Figure 10:**
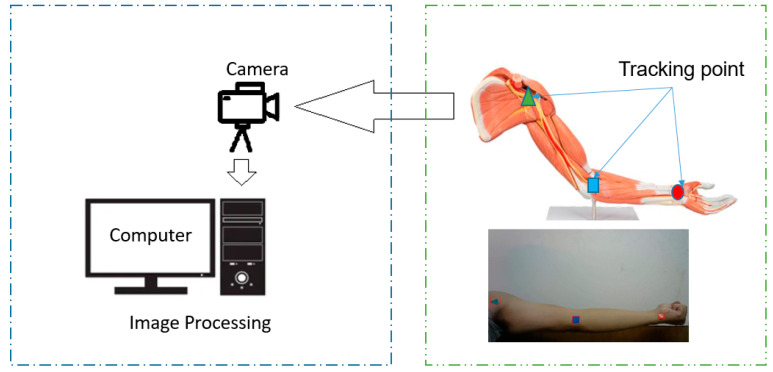
The experiment of image-processing tracking human joints.

The camera was used to verify the proposed algorithm. The results of the two methods can be compared to evaluate the efficiency of the proposed method. We used the same unit as the pixel to measure the location of the points on the joints. The X-Y plane was considered for the calculation in the experiment. Figure 10 shows the entire process of tracking the positions of the three points. Each point matched a markland with a different shape. We used three shapes to identify the three points in hand. When a person moves their hand, the camera receives a picture of the hand to determine the location of the markland. Coordinate origin selected the center point of the shoulder by tracking three points in a hand using the pyramid match algorithm.

## 4. Results and Discussions

### 4.1. IMU Signal Results at the Arm

**Figure 11:**
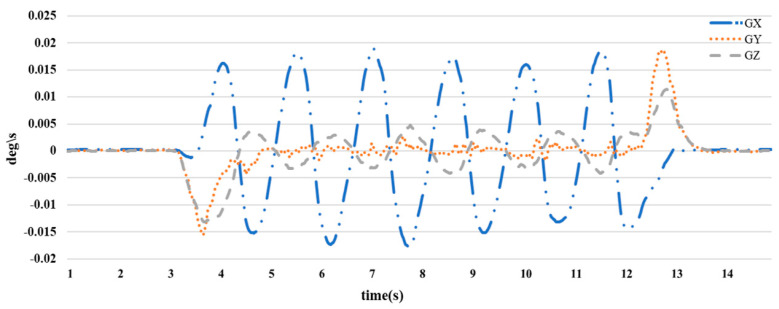
Angular velocity plot of arm movements.

When attaching an IMU sensor on the hand and collecting two signal charts of acceleration and angular velocity when the arm moves, we can make the following observations. Angular velocity signal: The angular velocity signal chart represents the rotation around the x, y, and z axes. When the arm moves, this chart will show peaks and valleys corresponding to phases of rotation and stopping during movement in Figure 11. Depending on the type of movement, the chart may have unique characteristics such as different rates and directions of rotation

**Figure 12:**
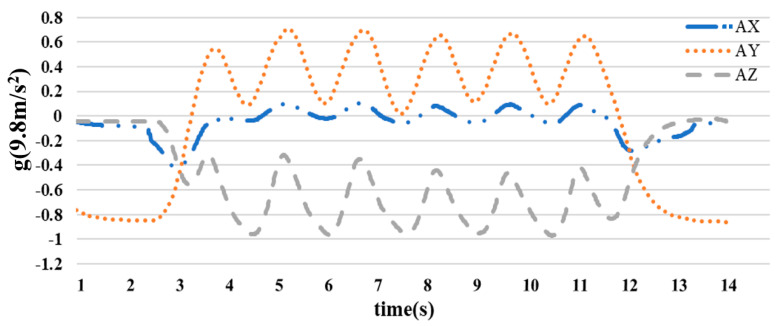
Acceleration plot of arm movements.

Acceleration signal: The acceleration signal chart represents the changes in velocity along the x, y, and z axes. When the arm moves, this chart will show peaks and valleys corresponding to phases of acceleration and deceleration during movement in Figure 12. Depending on the type of movement, the chart may have unique characteristics such as oscillations, rhythms, and varying rates of acceleration and deceleration.

We conducted a series of experiments to test our hypotheses and validate our mathematical model. The data collected from the experiments showed that our first hypothesis was largely accurate, with muscle contraction having little effect on the overall length of the human body. However, our second hypothesis proved to be more complex, with the relationship between the arm and body being both rotational and translational. To further investigate this relationship, we conducted additional experiments focusing specifically on the rotational and translational components. We found that the rotational relationship between the arm and body played a larger role in overall movement, particularly in activities that required a large range of motion, such as throwing a ball or swinging a bat. Our findings have significant implications for fields such as sports science and physical therapy. By understanding the relationship between muscle contraction and body length, as well as the rotational and translational components of arm movement, we can develop more effective rehabilitation and training programs. Additionally, our mathematical model for tracking arm motion can be applied in various contexts, such as motion capture for animation and virtual reality.

### 4.2. Results Observed on the X-Y Plane

**Figure 13:**
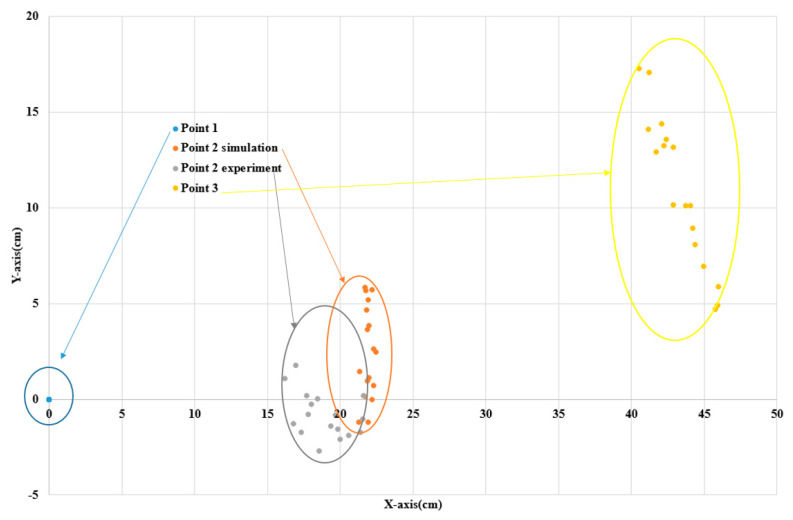
The distribution of center point of human joints on X-Y plane.

To assess the performance of the innovative approach, the experiment and simulation were compared in the X-Y plane, with centimeters as the unit of measurement. In the simulation, assuming that the coordinates of the point on the saddle joint (P3) are known, the location of a point on the hinge joint (P2) was determined. In the experiment, the location of each point was determined via image processing using a camera. Figure 13 presents the distributions of the three-point item, point 1, and point 3 (known at the beginning). However, point 2 of the simulation and experiment was found to have a slight difference.

**Figure 14:**
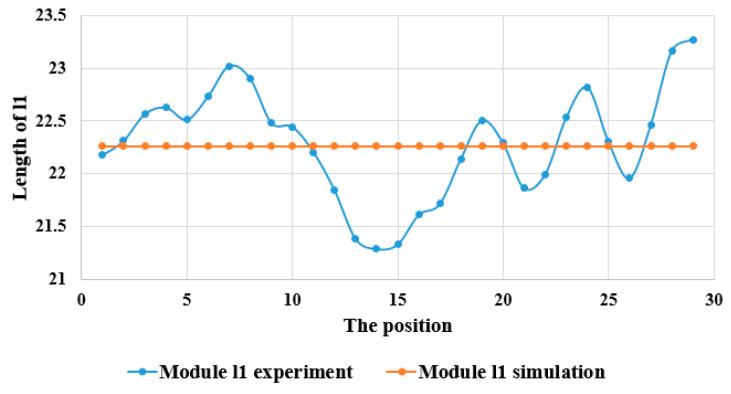
Comparison of distance of upper arm by using image processing and simulation.

**Figure 15:**
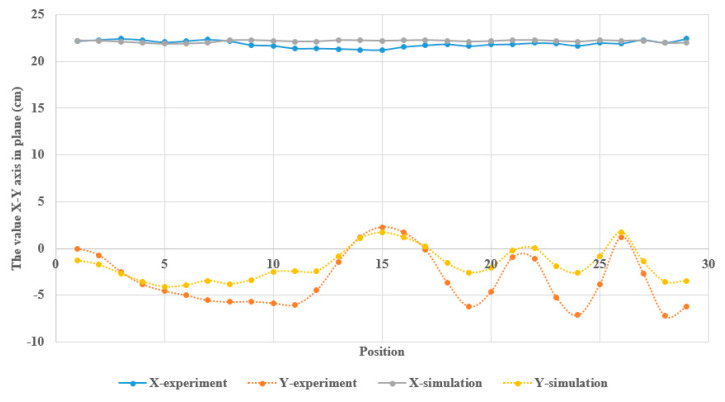
The location of the point 2 in simulation and experiment.

We obtained the results by conducting experiments and simulations at various locations. Figure 14 and Figure 15 display the results of l1 obtained through the simulations and experiments. The average peak deviation of the l1 error was 0.516 cm, which was due to contraction within the arm muscle. Additionally, the fluctuation observed in the l1 experiment reflected the squeezing of the hand muscles.

The standard deviations of the X-Y plane along the X and Y axes were 0.499 and 2.206 cm, respectively. The value of X remained constant and exhibited properties consistent with those of the simulation. However, the value of Y showed a discrepancy with the simulation owing to muscle contraction.

In Table 3, the Pearson correlation coefficient of −0.395746517 indicates a relatively weak and negative relationship between X-experiment and X-simulation. This suggests that as the values of X-experiment increase, the values of X-simulation tends to decrease, albeit weakly. However, the correlation coefficient does not reach a strong level of correlation, as the value is close to −0.4. Additionally, the Pearson correlation coefficient of 0.869627818 indicates a relatively strong and positive relationship between the Y-experiment and Y-simulation under consideration. The t-Statistic value of −3.452200841 indicates a significant difference between X-experiment and X-simulation. This indicates that the difference between the two variables is statistically significant and not due to random chance. The P(T ≤ t) two-tail value of 0.001784709 and 0.00002140844 indicates a very low probability of observing such a significant difference between X-experiment and X-simulation or Y-experiment and Y-simulation, respectively, by random chance alone.

## 5. Conclusions

In conclusion, our study demonstrates the effectiveness of using IMUs in accurately capturing human body movement posture. By optimizing the usage of IMUs and selecting a specific case to study and solve the problem, we were able to develop a systematic and highly applicable solution. This approach has potential applications in various fields, such as sports medicine and physical therapy, where accurate measurement of body movement is crucial. Our study will also demonstrate that joints can be monitored through the examination of IMU signals, and we will propose an innovative method for joint motion monitoring that will minimize the required number of IMU sensors, leading to cost savings and system simplicity. Individuals with high musculoskeletal health risks will benefit from an advanced algorithm for device joint monitoring, which will track and evaluate joint function in a comfortable and non-intrusive manner. This research will pave the way for a new approach to tracking human body movements, eliminating the need for cameras. Furthermore, we will further enhance the aspects of patient information security and professional ethics. Moreover, motion tracking is expected to become a widely adopted non-invasive procedure in future clinical practice.

## Figures and Tables

**Table 1:** The meanings of symbols in a formula.

Symbol	Meaning
k	Order of number of samples
W	Window size
a	$\mathrm{Accelerometer}\;\mathrm{data}\;\mathrm{from}\;\mathrm{IMU},a\in R^{W\times 3}$
ω	$\mathrm{Gyroscope}\;\mathrm{data}\;\mathrm{from}\;\mathrm{IMU},\omega \in R^{W\times 3}$
$\sigma_{a}^{2}$	The variances of the specific force rate measurements
$\sigma_{\omega }^{2}$	The variances of the specific angular rate measurements
$\bar{a}$	The per-channel mean of the specific force samples in W
$\left\|\right.\bar{a}\left.\right\|$	$\mathrm{Norm}\;\mathrm{of}\;\mathrm{mean}\;\bar{a}$
γ	The primary tuning parameter that has the largest effect on detection

**Table 2:** The meanings of symbols in geometry.

Symbol	Meaning
$C_{1}$	The orbit of the upper arm from two joints
$C_{2}$, $C_{2}^{’}$	The orbit of the arm from two joints
*x*_1_, *x*_2_, *y*_1_, *y*_2_	IMU position coordinates for positions 1 or 2 on the plane X-Y
*d*	The line in the plan X-Y with the relevant parameter
IMU	The device match on hand at center point of wrist
Yaw(α)	The angle is calculated from IMU
$l_{1}$	The distance from shoulder to center point of elbow
$l_{2}$	The distance from elbow to center point of wrist

**Table 3:** Statistical results between experiment and simulation.

	X-Experiment	X-Simulation	Y-Experiment	Y-Simulation
Mean	21.87910345	22.147143	−3.238344828	−1.735030819
Variance	0.12977131	0.0127978	8.155267448	3.064259288
Observations	29	29	29	29
Pearson Correlation	−0.395746517		0.869627818	
Hypothesized Mean Difference	0		0	
t Stat	−3.452200841		−5.09467942	
P(T ≤ t) two-tail	0.001784709		0.00002140844	
